# Anatomy and Pathology of Anterior and Lateral Hip Compartments Bursae: An Ultrasound Structured Approach in Sports Medicine

**DOI:** 10.3390/diagnostics16111731

**Published:** 2026-06-04

**Authors:** Antonio Corvino, Corrado Tagliati, Vincenzo Ricci, Fabio Corvino, Domenico Tafuri, Orlando Catalano, Giulio Cocco

**Affiliations:** 1 Medical, Movement and Wellbeing Sciences Department, University of Naples “Parthenope”, 80133 Naples, Italy; an.cor@hotmail.it (A.C.); domenico.tafuri@uniparthenope.it (D.T.); 2 AST Ancona, Ospedale di Comunità Maria Montessori di Chiaravalle, 60033 Chiaravalle, Italy; 3 Physical and Rehabilitation Medicine Unit, Luigi Sacco University Hospital, ASST Fatebenefratelli-Sacco, 20157 Milan, Italy; vincenzo.ricci58@gmail.com; 4 Vascular and Interventional Radiology Department, Cardarelli Hospital, 80131 Naples, Italy; effecorvino@gmail.com; 5 Radiology Unit, Istituto Diagnostico Varelli, 80126 Naples, Italy; orlando.catalano@istitutovarelli.it; 6 Department of Neuroscience, Imaging and Clinical Sciences, University “G. d’Annunzio”, 66100 Chieti, Italy; cocco.giulio@gmail.com

**Keywords:** hip bursae, bursitis, sports medicine, ultrasound

## Abstract

When assessing hip pain in athletes, it is important to focus on the extra-articular soft tissues that may clinically mimic joint pathology. One such extra-articular structure is the synovial bursa. Bursitis can clinically be misdiagnosed as joint-, tendon- or muscle-related pain. These pathological processes are a result of inflammation that is often secondary to acute trauma, overuse injuries, arthritis, or infection. Traumatic and overuse hip bursitis occur frequently in amateur and professional athletes and clinical presentation is often nonspecific. After clinical assessment, imaging plays an important role in diagnosis and in work-up of these lesions. Ultrasonography (US) is being increasingly used in the assessment of the hip because of the wide availability of US machines, the lower cost, and the unique real-time imaging capability which allows both static and dynamic evaluation as well as guidance of interventional procedures such as fluid aspiration and steroid injection. In order to obtain a correct diagnosis, an efficient US examination requires a thorough understanding of hip anatomy. In this setting, a structured approach based on identification of important US landmarks and compartmentalization of hip anatomy can significantly simplify the task. Sports physicians must be aware about the diagnostic and therapeutic possibilities offered by US in order to expedite rapid referral to a musculoskeletal specialist who can perform a point-of-care US examination of the hip by using a structured diagnostic approach. The purpose of this narrative review is to illustrate both the normal and pathological features of anterior and lateral hip compartments bursae using schematic diagrams and corresponding US images. We will concentrate on sport-related bursitis, of which we will discuss the etiology, clinic and principles of care management. Our aim is to promote the acquisition of these concepts in sports medicine in order to improve patient outcomes.

## 1. Introduction

Ultrasonographic evaluation of the hip is a powerful tool in the hands of a skilled operator. It allows a cost-effective targeted assessment based on patient symptoms, especially when combined with a concurrent physical examination. Moreover, ultrasound (US) has also been found to be useful in guiding diagnostic and therapeutic interventions. When compared with other imaging modalities, the benefits include lack of ionizing radiation, portability, speedy assessment, ability to compare findings with those of the contralateral side in a timely manner, and potential cost savings. Above all, the unique advantage of US over other imaging modalities is its real-time imaging capability, which allows evaluation when the operator applies compression with the probe or performs dynamic maneuvers, to evaluate for pain and reproducibility of symptoms with sonopalpation.

Conversely, US is limited in its ability to assess osteo-articular injuries about the hip such as fractures, femoro-acetabular impingement, labral tears, and articular cartilage injuries. These are generally more advantageously evaluated using radiography, computed tomography (CT), or magnetic resonance imaging (MRI), although in certain conditions US may allow acquisition of additional complementary findings [1,2].

An efficient US examination requires a thorough understanding of hip anatomy. In this setting, a structured approach based on identification of US landmarks and compartmentalization of the hip anatomy into anterior, lateral, and posterior aspects can significantly simplify the task [2,3,4].

For sports physicians, US is especially valuable in the assessment of hip injuries. In children and adolescents, 10–24% of sports-related injuries affect the hip as opposed to about 5–6% in adults [4,5].

In this narrative review intended primarily for sports physicians, we illustrate and describe both the normal and pathological features of bursae of anterior and lateral hip compartments assessed by using a structured US approach. We will concentrate on sport-related bursitis, of which we will discuss the etiology, clinic, imaging and principles of care management. To support these aims, we conducted a targeted literature search. We searched PubMed/MEDLINE, Scopus, Web of Science and Google Scholar for studies published from 1 January 1990 to 30 April 2026, assessing English language studies on imaging in anterior and lateral hip bursae and including results to the following publication types: clinical study; comparative study; controlled clinical trial; meta-analysis; multicenter study; observational study; randomized controlled trial; systematic review; case report; case series; narrative review.

## 2. Synovial Bursae and Pathological Conditions

Serous bursae consist of a synovial membrane enveloping a film of liquid. They are located at interfaces between moving structures, where friction must be reduced. Therefore, they are found between tendons and bones or between two tendons (subtendinous bursae); between muscles and tendons, bones, or ligaments (submuscular bursae); between aponeuroses and bones (subfascial bursae); and between cutaneous/subcutaneous tissues and bone, muscles, or tendons (subcutaneous bursae). They can also develop in areas where friction occurs (adventitial bursae) [6].

Some of these bursae are located near joints (non-communicating bursae); others are in direct communication with the joint cavity (communicating bursae). The main function of non-communicating bursae, located at the insertional areas of the anchor tendons of several joints, is to reduce the friction between tendon and bone. Communicating bursae, on the other hand, when an abundant intra-articular fluid collection occurs, function by reducing the joint cavity pressure, by expanding and being filled with the fluid coming from the cavity [7].

Bursae are lined with a synovial membrane. Histologically, the synovial membrane consists of two layers: superficial and deep. The cellular superficial layer is composed of synovial cells in an intercellular matrix, whereas the deep vascular layer consists of vascularized and fibrous connective tissue [6,7].

A bursal pathological condition can cause distention with abnormal fluid, synovial hypertrophy, or both. If a bursa communicates with a joint, then the bursal pathological condition may reflect the intra-articular process, such as joint effusion, synovial hypertrophy, and intra-articular bodies [7,8,9].

The term bursitis usually refers to sterile inflammation of the bursal cavities. The pathologic processes leading to bursitis may include direct trauma, overuse, crystal-induced arthropathies such as gout, inflammatory arthropathies such as rheumatoid arthritis, or infection in cases of septic bursitis [10,11] (Table 1).

## 3. General Ultrasound Features

US is probably the simplest, quickest, most cost-efficient imaging method to demonstrate a superficial distended bursa and to assess its internal architecture, size and relation on surrounding anatomical structures. Furthermore, US alone allows dynamic assessment of compressive effects on surrounding structures and abnormal motion of muscles and tendons. Thus, most authors recommend US as the primary imaging modality in the assessment of palpable masses near the joint or in patients with painful joints and inconclusive conventional radiographs. However, deep-seated bursae are better depicted on MRI or CT [12,13].

Under normal conditions, most bursae are collapsed and difficult to visualize on imaging studies. If the bursa is sufficiently distended, it can be visualized by US as the ultrasound beam is able to penetrate through this region [6,7].

In acute forms, bursal distention may be caused by simple fluid, which appears anechoic on US, or complex fluid or hemorrhage, which will have a heterogeneous appearance with variable echogenicity. In other cases, bursitis is chronic and is thus usually associated with synovial proliferation, wall thickening, and sometimes containing hyperechoic spots consistent with microcalcifications. Differentiation between complex fluid, e.g., clots and fibrin, and synovial hypertrophy may be difficult; on US, compressibility, lack of internal flow on color-Doppler and power-Doppler imaging, and movement or redistribution of contents with transducer pressure suggest complex fluid rather than synovial hypertrophy (Table 2). Rarely, tuberculosis was reported to cause bursitis; however, the latter is usually associated with other lesions, such as muscular abscesses, fistula formation and/or bone involvement [14,15,16].

However, musculoskeletal ultrasound is an operator-dependent imaging technique, with an inter-reader agreement of about 0.80. It is known that labral tears and articular cartilage injuries cannot be adequately assessed; however, dynamic evaluation and sonopalpation can be very useful in clinical practice [17,18].

## 4. Summary of Other Imaging Techniques and Relative Findings

In most cases, bursitis shows no abnormality on radiographs. The most common radiographic abnormality found is either bone irregularity or faint soft-tissue calcification. A distinct soft-tissue mass or opacity is generally not seen [6,7].

Bursitis usually appears as a low-density effusion or cyst on CT. Mean internal density measurements are typically less than 20 Hounsfield units. Septations and peripheral rim enhancement following intravenous contrast administration are sometimes observed. CT is particularly good at revealing bursal calcification due to gout, tuberculosis, synovial osteochondromatosis, or tumoral calcinosis [12].

Given its high soft-tissue contrast and multiplanar imaging capabilities, MRI is the modality of choice for evaluating both superficial and deep-seated bursae. MRI accurately demonstrates distention of the serous bursae by fluid or synovial proliferation. Typical features of bursitis are homogeneous low signal on T1-weighted images and homogeneous high signal on T2-weighted images, reflecting the simple fluid content of the bursa. If there are episodes of infection or hemorrhage, signal intensity becomes more variable and hyperintensity on T1-weighted sequences is commonly encountered. Synovial proliferation folds are well demonstrated on post-contrast MRI studies. Typically, there is no restricted diffusion within the bursa. Calcifications should be assessed on susceptibility weighted imaging or gradient echo images, although CT is more accurate in their detection. Also, MRI reliably assesses communication between the bursa and the joint as well as the anatomical relationship to surrounding structures. Marrow edema or bone irregularity, in addition to adjacent soft-tissue edema, can be detected [12,19]. The benefits of MR imaging are that it is less operator-dependent, can assess deep structures more confidently, and can cover a larger body part. However, the potential downside of MRI is that it is relatively more time-consuming and less available than US [6]. Moreover, MRI is an operator-dependent technique too, and tendon pathology and peritrochanteric edema on MRI are seen in both symptomatic and asymptomatic people [20,21,22].

## 5. Ultrasound Scanning and Hip Bursae

Technically, an array of transducers may be used for an adequate US assessment of the hip, depending on patient body habitus and the depth of the assessed structures. Broad-bandwidth high-frequency (7–15 MHz) linear probes may be employed to evaluate more superficial structures with adequate spatial resolution. Lower frequency (3–6 MHz) curvilinear probes allow better visualization of deep structures or in larger patients. Color- or power-Doppler techniques may be employed to differentiate vascular structures from neighboring anatomy or to assess the vascularity of identified disease. To facilitate US waves focusing on the most superficial layers, copious amounts of gel or a gel stand-off pad can be applied [23].

**Figure 1:**
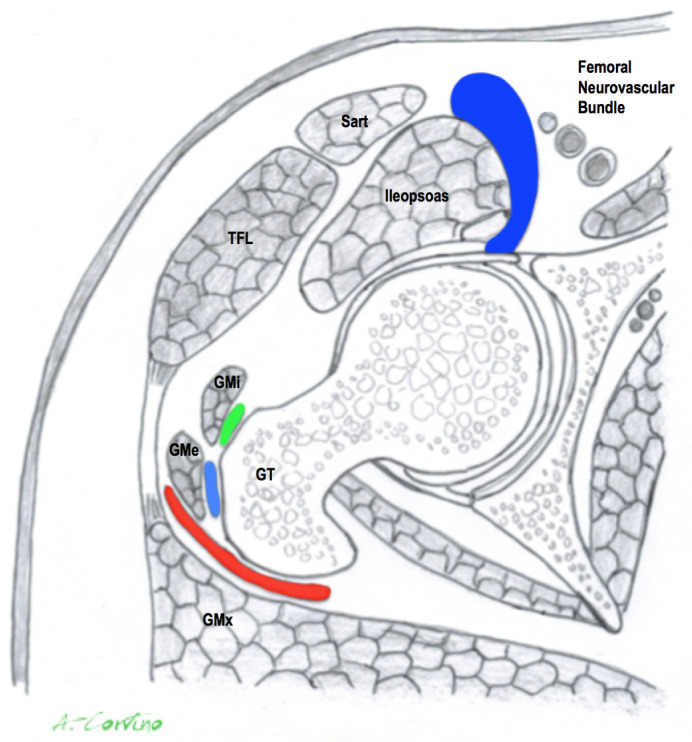
Hip bursae. Schematic transverse section through the hip joint shows iliopsoas bursa (dark blue), subgluteus minimus bursa (green), subgluteus medius bursa (light blue), and subgluteus maximus (or trochanteric) bursa (red). Sart sartorius, TFL tensor fascia latae, GMi gluteus minimus, GMe gluteus medius, GMx gluteus maximus, GT greater trochanter.

The routine scanning technique for US examination should consider the anterior, lateral and posterior aspects of the hip as separate quadrants [2]. According to this systematic US approach, the bursae around the hip are didactically divided into three groups: anterior (iliopsoas bursa), lateral (subgluteus maximus, subgluteus medius, piriformis, subgluteus minimus and gluteofemoral bursae), and posterior (obturator externus, obturator internus and ischial bursae) (Figure 1) [6]. This review will focus on the anterior and lateral hip compartments bursae.

## 6. Anterior Compartment

### 6.1. Iliopsoas Bursa

#### 6.1.1. Anatomic Key Points

The iliopsoas muscle complex is composed of two muscles with different areas of origin and the same distal insertion: psoas major muscle and iliacus muscle. The psoas major is a long fusiform muscle that originates on the vertebral bodies, transverse processes, and intervertebral disks of T12–L5. The iliacus is a triangular fan-shaped muscle that is composed of medial and lateral bundles and originates from the ventral lip of the iliac crest, superior two-thirds of the iliac fossa, and sacral ala. The psoas major and iliacus muscles converge at the level of the L5 to S2 vertebrae to form the iliopsoas muscle. Before this convergence, the psoas major tendon originates above the level of the inguinal ligament from within the center of the psoas major muscle. As the tendon courses distally, it rotates clockwise and migrates posteriorly within the muscle, lying immediately anterior to the hip joint, and inserts directly on the lesser trochanter. Common femoral artery and vein pass just medially to the fibers of the iliopsoas muscle in the so-called lacuna vasorum space, which constitute the medial compartment of the femoral triangle [24,25].

**Figure 2:**
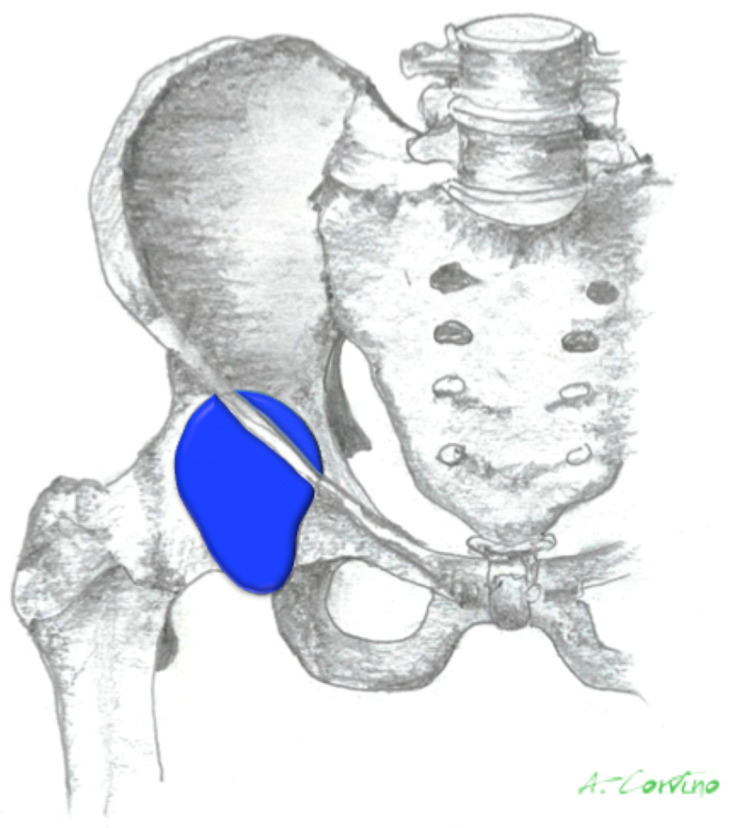
3D color illustration. The iliopsoas bursa (dark blue) is the largest bursa in the human body (on average, 5 to 7 cm long and 2 to 4 cm wide), presenting in 98% of individuals. It is situated between the musculotendinous portion of the iliopsoas tendon and the anterior capsule of the hip, bordered medially by the pectineus muscle and laterally by the anterior-inferior iliac spine. Communication between the iliopsoas bursa and hip joint by way of a defect between the pubofemoral and iliofemoral ligaments occurs in 15% of normal asymptomatic individuals.

The iliopsoas bursa is the largest bursa in the human body (on average, 5 to 7 cm long and 2 to 4 cm wide), presenting in 98% of individuals. It is situated beneath the musculotendinous portion of the iliopsoas, bordered medially by the pectineus muscle and laterally by the anterior-inferior iliac spine. Communication between the iliopsoas bursa and hip joint by way of a defect between the pubofemoral and iliofemoral ligaments occurs in 15% of normal asymptomatic individuals (Figure 2). In normal states, the bursa is collapsed and contains only a small amount of fluid. Its main function is to reduce the attrition between the iliopsoas tendon and the anterior aspect of the hip joint during muscle activation and joint movements [25,26].

#### 6.1.2. Diagnostic Landmark Approach

**Figure 3:**
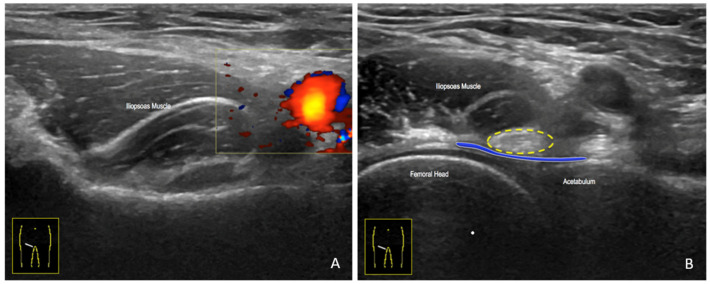
Probe position over the anterior hip at different transverse planes for the evaluation of the iliopsoas muscle, tendon and bursa. (**A**) Cranial US axial scan showing the muscle bellies of the iliopsoas, coursing in the lateral pelvis over the iliac bone, and the femoral neurovascular bundle with the femoral artery imaged on color-Doppler mode. (**B**) Caudal US axial scan which shows the iliopsoas muscle belly with its tendon in the typical eccentric position (dashed oval). This is the level to look for the iliopsoas bursa (dark blue), which intervenes between the tendon and the anterior capsule of hip.

**Figure 4:**
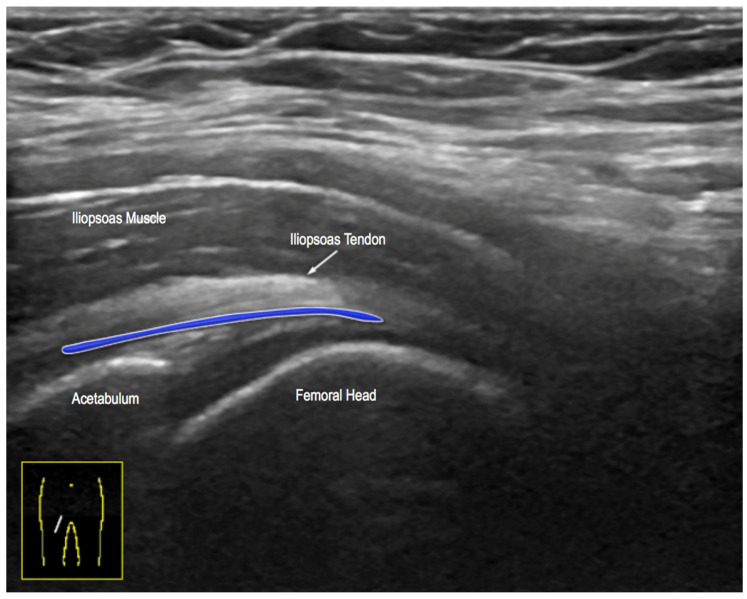
Probe position in a longitudinal oblique scan, over the anterior aspect of the hip joint. This image shows the relationship between the iliopsoas muscle and tendon, seen in a longitudinal plane, and the underlying hip joint with acetabulum and femoral head. This is the level to look for the iliopsoas bursa (dark blue), which intervenes between the tendon and the anterior capsule of hip.

The patient is lying supine on the table with the leg extended and the hip slightly extrarotated. Palpate the antero-inferior iliac spine and position the probe just medially to it in a transverse plane. At this level, the US image shows, from lateral to medial, the hyperechoic cortex of the iliac bone with the attachment of the rectus femoris tendon, the fibers of the iliac muscle, the fibers of the psoas major muscle and the femoral neurovascular bundle. Maintaining a transverse orientation, it is possible to follow the iliopsoas muscle moving the transducer from cranial to caudal positions: on the US image, the myotendinous junction of the iliopsoas muscle can be progressively seen forming by the two distinct muscular bellies until the hyperechoic fibrillar oval structure of the tendon appears in a postero-medial eccentric position (Figure 3A,B). At this level, turn the probe by 90° and follow the tendon along its long axis until its insertion on the lesser trochanter. The longitudinal US scan shows the cortex of the femoral head covered by the articular cartilage and the anterior joint capsule (normally the anterior joint recess is a virtual space); the acetabulum is located proximally, covered by the iliopsoas tendon and the rectus femoris muscle fibers (Figure 4). This is the level to look for the iliopsoas bursa, which intervenes between the tendon and the anterior capsule of the hip; it is collapsed in normal states and, therefore, it is difficult to be detected with US (Figure 3B and Figure 4) [27,28].

### 6.2. Iliopsoas Bursitis

The three main causes of iliopsoas bursitis (IB) are acute trauma, overuse injury, and rheumatoid arthritis. Other disease processes that involve the bursa are hip osteoarthritis, pigmented villonodular synovitis, and synovial osteochondromatosis. IB is also idiopathic [29,30].

It has been proposed that acute or chronic occupational trauma and sports injuries have accounted for the majority of reported cases of IB. Overuse is the most common cause of IB in the athletic active population. Bursitis incurred during sporting activity is likely to be the result of overuse trauma imparted to the bursa during hip flexion and extension. Specifically, it is the result of constant friction from the overlying iliopsoas tendon that can be caused by the repetitive chronic injuries. Thus, IB is commonly seen in individuals participating in strength training, rowing, uphill running, ballet, jumping, and competitive track and field [29,30,31].

Patients with IB present with inguinal pain and an audible or palpable snap that can be reproduced by aggravating activities. Physical examination findings include tenderness to palpation, tenderness in the femoral triangle, weak external rotation strength in hip flexion, a positive Thomas test, and a positive snapping hip sign. IB can be asymptomatic. Various other symptoms related to compression of adjacent structures should also be considered. In the groin, pressure on the femoral vein can cause outflow impairment, limb edema, and, possibly, venous thrombosis, whereas excessive pressure on the femoral nerve can cause neural impairment. Compression of various intrapelvic structures has also been described [32].

**Figure 5:**
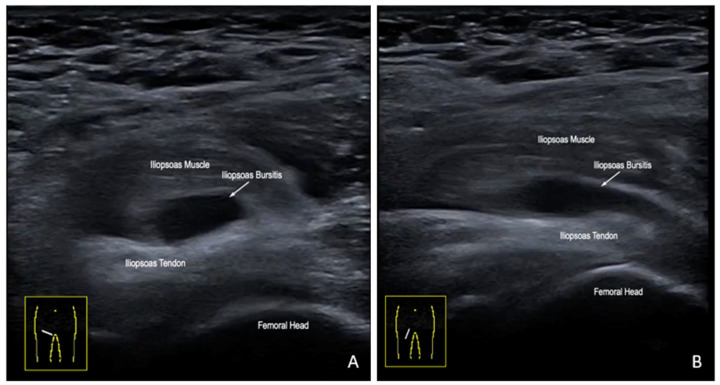
Overuse iliopsoas bursitis. Ultrasound images obtained over the femoral head in the transverse (**A**) and longitudinal sagittal (**B**) planes demonstrate a well-defined anechoic fluid distention of the iliopsoas bursa, located deep to the muscular portion of the iliopsoas, consistent with bursitis. Mild thickening of the adjacent iliopsoas tendon suggests associated early tendinopathic changes.

The correct diagnosis of an IB is usually made by US following the fluid-filled structure on successive axial images, recognizing that it is contiguous with the iliopsoas tendon (Figure 5A,B) [33].

Furthermore, when distended by a considerable amount of fluid, iliopsoas bursa takes on lobulated outlines showing a “balance weight” or “heart” shape in the axial plane. This diagnostic aspect is characteristic [34]. MRI can be more sensitive than US for the detection of iliopsoas bursitis (60% vs. 53%) [35].

The main differential diagnoses include paralabral cyst, ganglion cyst, iliopsoas musculotendinous strain or rupture, arterial pseudoaneurysms and inguinal lymphadenopathies [36,37] (Table 3).

Treatment of iliopsoas bursitis should begin with conservative measures, including a program of rest, physical therapy for iliopsoas stretching, and oral anti-inflammatories. If these measures fail, steroid injection should be attempted under US or fluoroscopy [38,39,40]. Steroid injection has been shown to provide permanent relief in 50% of the population and 2 to 8 months of relief otherwise. If conservative measures fail, surgery should be considered. Surgical treatments include partial release and lengthening of the iliopsoas tendon, with 50% to 90% success rates. Complications of surgery include hip flexion weakness, hematoma, skin paresthesia, and recurrence of symptoms [32].

## 7. Lateral Compartment

### 7.1. Subgluteus Maximus, Medius and Minimus Bursae

#### 7.1.1. Anatomic Key Points

Gluteal muscles are composed of three structures disposed into two layers: gluteus maximus superficially and gluteus medius and gluteus minimus more deeply. The gluteus maximus is the largest and most superficial of gluteus muscles; it is a thick flat sheet of muscle that forms the contour of the buttock. It arises from the gluteal line on the posterior border of the ilium, from the side of the lower sacrum and coccyx, from the sacrotuberous ligament and from the aponeurosis of the erector spinae. Gluteus maximus fibers cover superficially the entire area comprising the ilium, the sacrum and the ischium, to reach the ilio-tibial tract and the femur laterally: the muscle does not have an attachment on the greater trochanter but courses superficially over the posterior facet and is separated from it by the trochanteric bursa. Its superior fibers insert into the posterior aspect of the iliotibial band, whereas the inferior fibers end on the gluteal tuberosity located at the linea aspera.

The gluteus medius is a deep muscle: its posterior third is located deep to the gluteus maximus muscle, whereas its anterior two thirds are more superficial, just below the fascia latae. The gluteus medius originates from the posterior two thirds of the iliac wing and then courses laterally and downwards. It inserts into the lateral facet of the greater trochanter with its more posterior portion and onto the superolateral aspect of the greater trochanter with its middle-anterior portion.

The gluteus minimus is the deepest gluteal muscle; it takes origin from the anterior third of the posterior iliac wing and, with its fibers, comes laterally and downwards to attach onto the anterior facet of the greater trochanter of the femur [41,42].

**Figure 6:**
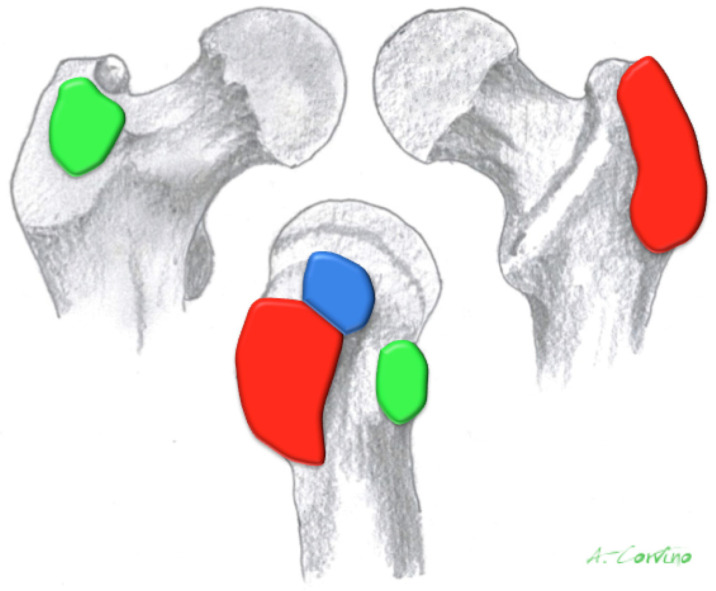
3D color illustration. Location of the three greater trochanter bursae, the trochanteric or subgluteus maximus bursa (red), the subgluteus medius bursa (light blue), and the subgluteus minimus bursa (green).

Several synovial bursae are located around the greater trochanter allowing a smooth gliding of the tendons and fasciae latae against the bone. The most important are the trochanteric bursa and the bursae of the gluteus medius and minimus. The trochanteric bursa is the largest and the most constantly present bursa around the greater trochanter. It separates the undersurface of the gluteus maximus and the fasciae latae from the tendon of the gluteus medius and the lateral aspect of the greater trochanter. More distally, the subgluteus maximus bursa lies between the distal attachment of the gluteus maximus muscle and the dorsal aspect of the femur. The bursa of the gluteus medius, which is commonly referred to as the subgluteus medius bursa, is located between the anterosuperior part of the lateral facet of the greater trochanter and the gluteus medius tendon, whereas the gluteus minimus bursa, also named the subgluteus minimus bursa, is found anteromedially to the insertion of the gluteus minimus (Figure 6) [6,7].

#### 7.1.2. Diagnostic Landmark Approach

**Figure 7:**
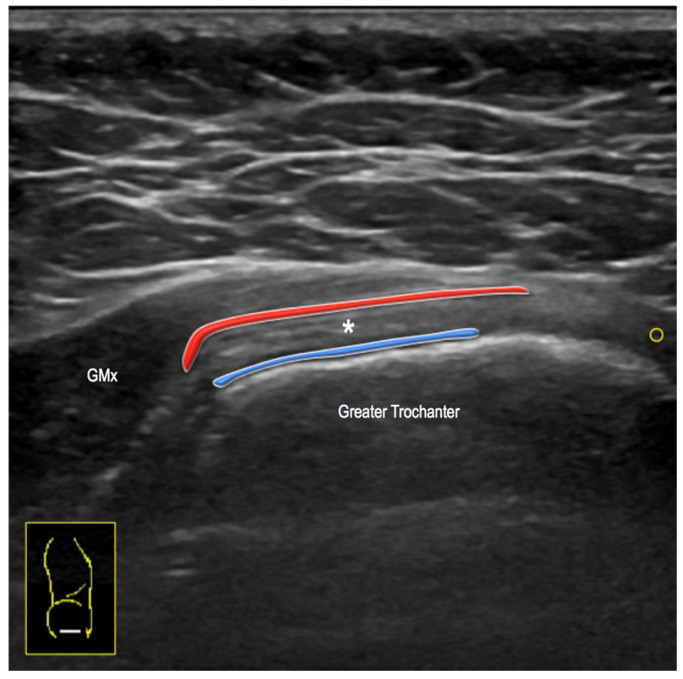
Probe position over the lateral hip for the evaluation of the gluteal muscles, tendons and bursae. Corresponding US axial scan over the greater trochanter shows the “cuff-like” appearance of gluteal muscle insertions on the greater trochanter; the gluteus minimus tendon (yellow circle) attaches on the anterior aspect of the trochanter, gluteus medius tendon (asterisk) is positioned in the middle, gluteus maximus tendon covers the trochanter with its muscular fibers (Gmx). Due to too small a fluid content, the bursae around the greater trochanter are not easily visible or identifiable with US in normal conditions. The trochanteric bursa (red) separates the undersurface of the gluteus maximus and the fasciae latae from the tendon of the gluteus medius and the lateral aspect of the greater trochanter. The bursa of the gluteus medius (light blue) is located between the anterosuperior part of the lateral facet of the greater trochanter and the gluteus medius tendon.

The patient lies on the table in lateral decubitus. Start the evaluation of the gluteal region palpating the lateral aspect of the hip and find the bony prominence of the greater trochanter of the femur. The greater trochanter of the femur is, in fact, an essential osseous landmark of the lateral hip region, with important muscular attachments, as well as bursal and aponeurotic structures in its vicinity. It consists of four facets: anterior, lateral, superolateral, and posterior. Place the probe with a transverse orientation over the greater trochanter: the US image shows the typical “rotator cuff appearance” of the tendinous insertions of the gluteal muscles covered by the muscular fibers of the gluteus maximus. Proceeding from anterior to posterior along the hyperechoic cortex of the greater trochanter there are three different hyperechoic fibrillar structures: the tendon of the gluteus minimus (inserting onto the anterior facet of the trochanter), the anterior portion of the gluteus medius tendon (inserting on the lateral facet of the trochanter) and the posterior portion of the tendon of the gluteus medius (on the superolateral facet of the trochanter), covered by gluteus maximus muscular fibers. Then, rotate the probe by 90° and slide the transducer from anterior to posterior to assess each tendon along its long axis. Fascia latae, which courses between gluteus maximus posteriorly and tensor fasciae latae anteriorly, could be used as an important anatomic landmark during the US examination. It is indeed detectable on the US image as a hyperechoic layer of fibrous tissue standing just below the subcutaneous fat: remember that gluteus medius and gluteus minimus course under that fascia. Due to too small a fluid content, the bursae around the greater trochanter are not easily visible identifiable with US in normal conditions (Figure 7) [28,29].

### 7.2. Trochanteric Bursitis

Symptoms referring to the greater trochanter are common in athletes, and the condition is often referred to as greater trochanteric pain syndrome (GTPS). In this setting, contrary to what is often assumed, bursal distention is uncommon; in fact, it is reported in about 20% of patients. In addition, it has been shown that the bursa in these cases is typically not inflamed and not a primary cause of symptoms. The underlying pathological condition is most commonly related to tendinosis (reported in about 50% of patients) and, rarely, partial or full thickness tears of the gluteus minimus and medius tendons. Therefore, the continuing use of the term “trochanteric bursitis” is perhaps inappropriate [37,43], as GTPS has a multifactorial nature, such as tendon pathology, bursitis and iliotibial band abnormalities [44].

Similarly to bursitis of other body sites, trochanteric bursae can become inflamed from a variety of different sources, including trauma and repetitive motion. In athletes, causes include a tight iliotibial band, which may be a precursor to external snapping hip syndrome. Athletes with wide pelvises or excessive foot pronation are predisposed to trochanteric bursitis. Patients with a leg-length discrepancy or runners that train on baked surfaces or road cambers are also at increased risk. Repetitive trauma, such as the continuously falling ice skater, may cause chronic bursitis [45].

The clinical symptoms are varied. All patients complain of aching over the trochanteric area and lateral thigh. This pain may be acute at the onset, or it may build up gradually over time, sometimes lasting for many months or even years. In chronic cases, discomfort may be vague, and it may be difficult to describe its exact location. Some patients may have difficulty walking or walk with a limp. The pain experienced with trochanteric bursitis can radiate distally and must be distinguished from that associated with lumbosacral spine disease. Other neuromuscular symptoms must be ruled out through history and physical examination. On physical examination, the patient will have direct tenderness to palpation over the greater trochanter. Pain is exaggerated by resisted abduction and external rotation. Pain is also elicited by asking the patient to stand from a squatted position with the hips internally rotated [32].

**Figure 8:**
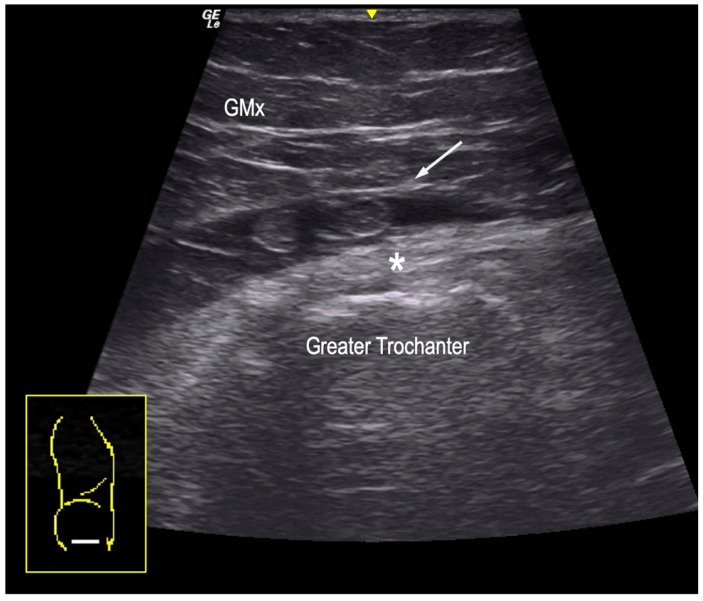
Traumatic trochanteric bursitis. A 24-year-old woman, volleyball player, with subgluteus maximus bursal bursitis. Short-axis US image of the lateral hip shows hypoechoic distention of the subgluteus maximus or trochanteric bursa (arrow) lying deep to the gluteus maximus (Gmx), and superficial to the greater trochanter and gluteus medius tendon (asterisk). Internal rounded clots can be appreciated inside the bursa. Bursal walls are not thickened because the pathologic process was due to an acute traumatic event.

In GTPS, US has been reported as a useful and accurate means to assess pathologic changes in the gluteus medius and minimus tendons, a condition referred to as “rotator cuff tear of the hip”, as well as the bursal pathology. Fluid distension of the trochanteric bursa appears as a well-circumscribed crescentic-shaped hypoechoic to anechoic collection located superficially to the posterior insertion of the gluteus medius and the lateral aspect of the greater trochanter and deep to the gluteus maximus (Figure 8). US and MRI show similar accuracy in trochanteric bursitis assessment [35].

From the pathophysiologic point of view, trochanteric bursitis could be interpreted as a true impingement syndrome. When the hip abductor tendons dysfunction or tear, disuse atrophy of the involved muscles may lead to loss of containment of the femoral head, lateral subluxation and impingement of the greater trochanter on the fasciae latae, thus sustaining development of bursitis [37,46].

Apart from bursitis, the main imaging features and differential diagnoses of GTPS are reported in Table 4 [47,48].

Treatment of trochanteric bursitis should proceed in a stepwise fashion. Despite the high prevalence of this condition, there are no controlled studies in regard to treatment protocols. Any offending activities should be removed from the patient’s training program. A physical therapy program focused on gluteal strengthening, iliotibial band stretching, and proper body mechanics should be implemented. In fact, targeted physiotherapy including load management, tendon rehabilitation, and gluteal strengthening showed long-term benefits for most patients, although improvements may not be immediate. However, oral anti-inflammatories and steroid injections should be considered as they can offer short-term relief. In particular, combining PT with CSI appears to be more effective in managing acute symptoms than targeted physiotherapy alone [49,50]. Athletes may return to play once symptoms have resolved. Surgical intervention should be considered for patients with refractory symptoms. Bursectomies, bone débridement, and tendon release have been attempted. One study reported excellent success rates and patients returning to full activity after longitudinal release of the iliotibial band and excision of the trochanteric bursa [32].

## 8. Conclusions

Familiarity with the normal anatomy, pathology, and imaging characteristics of bursae is important as bursitis can mimic pain related to joints, periarticular tendons, and muscles. Distinguishing bursitis from other causes of pain will direct the clinician toward focused management of bursitis, including image-guided therapeutic injections utilizing steroids and long-acting local anesthetics. In this setting, US can in many cases provide all the information requested, from diagnosis to care management, thus avoiding other in-depth but time-consuming and more expensive radiologic procedures, such as MRI. The novelty of this manuscript relies on the proposed structured ultrasound approach and its sports medicine perspective. We hope that the acquisition of these concepts in sports medicine could help in improving patient outcomes. Future studies will need to evaluate if ultrasound technological advancements with portable devices improving accessibility could reduce MRI dependence, taking into account the impelling necessity of a green radiology.

## Figures and Tables

**Table 1:** Pathologic processes of bursae.

Category	Specific Clinical Diagnosis
Trauma	Repetitive injury or acute trauma
Inflammation	Rheumatoid arthritis
	Gout
	Calcium hydroxyapatite deposition
	Infection
Synovial Proliferation	Pigmented villonodular synovitis
	Primary synovial chondromatosis
	Lipoma arborescens
	Amyloidosis

**Table 2:** Types of bursal involvement.

Category	Features
Acute bursitis	Homogeneous content, smooth and thin synovia
Subacute hematoma	Inhomogeneous content, moving clots
Chronic bursitis	Inhomogeneous content, synovial hypertrophy
Tuberculosis	Bursitis with muscular abscesses, fistula formation and bone involvement

**Table 3:** Iliopsoas bursitis differential diagnosis.

Disease	Features
Iliopsoas musculotendinous strain or rupture	Tendon inhomogeneity and discontinuity
Paralabral cyst	Small cystic lesions lateral to the iliopsoas related to labral tear
Ganglion cyst	Synovial cystic mass related to microtrauma
Arterial pseudoaneurysms	Lesion with Doppler swirling blood flow signal
Inguinal lymphadenopathies	Solid ovoid masses with internal vascularity

**Table 4:** Lateral compartment bursitis differential diagnosis.

Disease	Features
Gluteal tendinosis	Hypoechoic tendon thickening
Gluteal tendon injuries	Tendon discontinuity, adjacent fluid
Proximal iliotibial band syndrome	Thickening and hypo-echogenicity of the iliotibial band origin
Hydroxyapatite deposition disease	Calcific tendinitis
Morel-Lavallée lesion	Fluid collection due to a shearing of subcutaneous tissues away from underlying fascia

## Data Availability

No new data were created or analyzed in this study. Data sharing is not applicable to this article.

## Abbreviations

The following abbreviations are used in this manuscript:

US	Ultrasound
CT	Computed tomography
MRI	Magnetic resonance imaging
IB	Iliopsoas bursitis
GTPS	Greater trochanteric pain syndrome
